# Rapamycin Ameliorates Kidney Fibrosis by Inhibiting the Activation of mTOR Signaling in Interstitial Macrophages and Myofibroblasts

**DOI:** 10.1371/journal.pone.0033626

**Published:** 2012-03-28

**Authors:** Guochun Chen, Huihui Chen, Chang Wang, Youming Peng, Lin Sun, Hong Liu, Fuyou Liu

**Affiliations:** 1 Department of Nephrology, the Second Xiangya Hospital, Central South University, Changsha, Hunan, P.R. China; 2 Kidney Research Institute of Central South University, Changsha, Hunan, P.R. China; 3 Department of Ophthalmology, the Second Xiangya Hospital, Central South University, Changsha, Hunan, P.R. China; French National Centre for Scientific Research, France

## Abstract

Interstitial fibrosis is an inevitable outcome of all kinds of progressive chronic kidney disease (CKD). Emerging data indicate that rapamycin can ameliorate kidney fibrosis by reducing the interstitial infiltrates and accumulation of extra cellular matrix (ECM). However, the cellular mechanism that regulates those changes has not been well understood yet. In this study, we revealed the persistent activation of mammalian target of rapamycin (mTOR) signaling in the interstitial macrophages and myofibroblasts, but rarely in injured proximal epithelial cells, CD4+ T cells, neutrophils, or endothelial cells, during the development of kidney fibrosis. Administration of rapamycin to unilateral ureteral obstruction (UUO) mice significantly suppressed the immunoreactivity of mTOR signaling, which decreased the inflammatory responses and ECM accumulation in the obstructed kidneys. Isolated macrophages from rapamycin-treated obstructed kidneys presented less inflammatory activity than vehicle groups. In vitro study confirmed that rapamycin significantly inhibited the fibrogenic activation of cultured fibroblasts (NIH3T3 cells), which was induced by the stimulation of TGF-β_1_. Further experiment revealed that rapamycin did not directly inhibit the fibrogenesis of HK2 cells with aristolochic acid treatment. Our findings clarified that rapamycin can ameliorate kidney fibrosis by blocking the mTOR signaling in interstitial macrophages and myofibroblasts.

## Introduction

Tubulointerstitial fibrosis is the final common pathway of a wide variety of chronic progressive kidney diseases. Intense studies have focused on the molecular and cellular pathogenesis of interstitial fibrosis due to the strong correlation between the degree of interstitial fibrosis and renal functional loss in CKD. Recently, studies in a wide variety of animal models confirmed that treatment of rapamycin to inhibit mTOR could markedly ameliorate the interstitial inflammation, fibrosis, and loss of renal function associated with CKD [1]–[7]. However, little has been clarified in these studies upon the cellular targets of rapamycin, regarding its protective role in kidney fibrosis.

Progression of renal fibrosis can initially be characterized as induction of inflammatory response and ultimately result in widespread fibrotic changes. Multiple cell types within the interstitium, including kidney resident cells and infiltrates from circulation, directly contribute to the induction of inflammatory cascade and the fibrogenic process as a source of various proinflammatory and profibrotic molecules [8]–[10]. To date, the regulatory mechanism in these effector cells still remains obscure in kidney fibrosis, which limits the prevention and early interruption in the disease development.

mTOR is a major effector of cell growth and protein synthesis via the direct functional control of its downstream targets, ribosomal protein S6 kinase (S6k) and eukaryotic initiation factor 4E-binding protein-1 (4EBP-1) [11]. Recently, novel regulation of mTOR signaling has been identified in various pathological conditions, including activation of macrophages [12], [13] and myofibroblasts [14]–[16], indicating the importance of mTOR in the regulation of kidney fibrosis. However, it is unclear which cell types have mTRO activation and where rapamycin works on during the development of kidney fibrosis.

In this study, we looked into each specific cell type in the kidney to evaluate the role of rapamycin in renal fibrosis. We characterized the activation pattern of mTOR signaling in different renal cell types during kidney injury-fibrosis; we also evaluated the effect of rapamycin on the fibrogenic activity of cultured fibroblasts, HK2 cells and macrophages isolated from the fibrotic kidneys.

## Materials and Methods

### Ethics statement

All experiments were performed in accordance with the animal experimental guidelines issued by the Animal Care and Use Committee at Xiangya Medical School of Central South University. This study was approved by the Animal Care and Use Committee of the 2^nd^ Xiangya Hospital (protocol approval number 2008-S 062).

### Animals

C57BL/6 mice were obtained from the animal facility in the 2^nd^ Xiangya hospital and maintained under specific pathogen-free conditions. Rapamycin (2 mg/kg·day) (LC laboratories, Woburn, USA) was administered to a subgroup of UUO mice by daily intraperitoneal injections starting one day prior to surgery and continuing until termination of the experiment.

### Induction of kidney injury in mice

Female C57BL/6 mice aged 8–10 weeks weighing 20–22 g were used for induction of kidney injury. In brief, ischemia-reperfusion-injury (IRI) was induced by the retroperitoneal approach on both kidneys for 28 min at 37°C (moderate IRI). One milliliter of warm saline (37°C) was injected intraperitoneally after surgery for volume supplement. Sham operations were performed with exposure of both kidneys but without induction of ischemia. To generate the UUO mice, the left kidney and ureter were exposed via a flank incision. The ureter was ligated at two points proximal to the kidney with 6–0 silk. The wound was closed in layers. Sham animals had kidney exposed but ureter was not tied.

### Kidney tissue preparation

Mice were anesthetized, sacrificed and immediatlely perfused via the left ventricle with ice-cold PBS for 2 min. Kidneys were hemi-sectioned and portions were snap frozen in liquid nitrogen for later western blot and real-time qPCR analysis. Some kidneys were fixed in 10% neutral buffered formalin at 4°C for 12 hr, processed, embedded in paraffin wax, sectioned in 4 µm and stored at room temperature for use. Some kidneys were fixed in 4% PLP fixative (4% paraformaldehyde, 75 mM L-lysine, 10 mM sodium periodate) for 4 hr at 4°C, cryoprotected in 30% sucrose and snap frozen in optimal cutting temperature (OCT, Sakura FineTek). Frozen kidneys were sectioned in 7 µm for immunofluorescent stainging.

### Renal histological analysis

Kidney paraffin sections were stained with hematoxylin-eosin (HE) using standard procedures. HE-stained paraffin sections were assessed by quantitative measurement of tubular injury in 10 individual high-power fields (magnification ×400) per kidney. A percentage of the area affected was estimated for the number of necrotic cells, loss of brush border, cast formation, and tubule dilation as follows: 0, 0 to 5%; 1, 5 to 10%; 2, 11 to 25%; 3, 26 to 45%; 4, 46 to 75%, and 5, >76%. The matrix score for collagen-I deposition in the renal cortical interstitium was determined by procedures in accordance with previous reports [17]. The fields analyzed in each section were selected randomly. Ten separate, nonoverlapping microscopic fields of each kidney section were averaged to yield the score of each kidney. The scores for 3–6 separate animals for each group were then averaged.

### Immunofluorescence and immunohistochemical staining

All stainings of kidney were performed on 4 µm paraffin setions or 7 µm cryosections as previously described [18]. In brief, cryosections were air-dried for 15 min, then primary antibodies against the following proteins were used: pS6K (rabbit, 1∶100, Cell signaling, USA), F4/80 (rat, 1∶100, Abcam, USA), CD3 (rabbit 1∶100, Vectorlab, USA), CD4 (mouse, 1∶100, Abcam, USA), anti-neutrophil (rat, 1∶50, Santa Cruz, USA), αSMA (mouse, 1∶200, Abcam, USA), collagen-I (rabbit, 1∶500, Abcam, USA), Kim-1 (goat, 1∶100, R&D, USA), Lotus tetragonolobus lectin (LTL, 1∶1000, Vectorlab, USA). The slides were then exposed to FITC (1∶200) or Cy3-labeled (1∶500) secondary antibodies (Jackson ImmunoResearch, USA). Sections were mounted in Vectashield medium containing DAPI (Invitrogen, USA). Representative images were taken with confocal microscopes (Leica TCS SP5).

Immunohistochemical stains were performed on formalin fixed, paraffin embedded 4 µm sections. Sections were rehydrated and antigens retrieved using heated citrate. Incubation of primary antibodies was performed same as decribed above. Staining was visualized using horseradish-peroxidase coupled secondary antibodies (Vectastain elite, Vector Labs).

Related isotype immunoglobulins (Jackson ImmunoResearch, USA) were used as negative controls in all stainings. All immunohistochemical analyses were repeated at least three times and representative images were presented.

### Isolation of F4/80+ macrophges from obstructed kidneys

At day 1 post-obstruction, kidneys derived from either vehicle or rapamycin-treated groups were harvested, minced, and homogenized, followed by incubation with 0.1% collagenase (Worthington, USA) and 20 g/ml DNase I (Qiagen, USA) for 30 min at 37°C. Following the manufacture's instruction, mononuclear cells were obtained by density separation using Lympholyte M (Cedarlane, USA). Macrophages were labeled with biotin-conjugated rat anti-mouse F4/80, purified and enriched using MACS (Miltenyi Biotec). Total RNA was extracted from enriched F4/80 macrophages and reverse transcribed for real-time qPCR analysis of gene expression.

### Cell culture and immunocytochemical staining

The NIH 3T3 fibroblast cell line and HK2 cell line from ATCC (American Type Culture Collection) were cultured in DMEM medium supplemented with 10% FBS until the cells were 80% confluent. Cells were then incubated in DMEM medium containing 0.2% FBS for 24 hr. NIH3T3 cells and HK2 cells were cultured for 24 to 48 hr in the presence of 10 ng/ml recombinant human TGF-β_1_ (PeproTech, NJ, USA) or 5 µg/ml aristolochic acid (AA, Sigma), with or without addition of 50 nmol rapamycin (LC lab, MA, USA) for 12 hr. Portions of NIH3T3 cells and HK2 cells were cultured in four-chamber glass and immunocytochemical staining was performed as previously described [18]. In brief, Cells were fixed and blocked before immunocytochemical staining. Cells were then incubated in primary antibodies (including αSMA and pS6K) at 4°C overnight, followed by incubation in secondary antibodies consisting of anti-rabbit-Cy3 and anti-mouse-FITC. After rinsing in PBS, slides were mounted with Vectashield mounting medium containing DAPI (Invitrogen) and visualized under a confocal microscope (Leica TCS SP5). All immunocytochemical analyses were repeated 3 or more times and related isotype immunoglobulins were used as negative controls.

### Western blot analysis

Lysates of kidney or cultured cells were prepared as previously described [19]. Membranes were incubated with the following primary antibodies, respectively: rabbit antibody to p-mTOR and pS6K (Cell signaling, 1 in 1000), mouse antibody to αSMA and vimentin (Abcam, 1∶1000), rabbit antibody to collagen-I (Abcam, 1∶1000). β-actin–specific antibody (Abcam, 1∶1000) was used for loading controls on stripped membranes. Horseradish peroxidase–conjugated secondary antibodies were applied, and enhanced chemiluminescence (Thermo, IL, USA) was used to visualize bands.

### Evaluation of mRNA expression by real-time qPCR

To determine the gene expression profiles of kidney tissues, real-time qPCR was performed as previously described [19], to compare designated mRNA expression of obstructive kidneys in different groups. In brief, total RNA was extracted from the kidney cortexes using an RNA isolation kit (Qiagen, RNeasy Mini Kit). To ensure samples without genomic DNA contamination, total RNA was treated with DNase (Qiagen, RNase-Free DNase Set) and cDNA was synthesized using a Synthesis Kit (Bio-rad, USA). Total cDNA (1 µl) was loaded in each well, mixed with PCR master mix (TaqMan Universal, Applied Biosystems, USA) and pre-designed primers (IDT, San Diego, USA) for TNF-α, IL-1β, CXCL-1, MCP-1, TGF-β_1_ CTGF and Col1α2, respectively (Listed in Table 1). The procedure for real-time qPCR included 2 min at 50°C, 15 min at 95°C, followed by 40 cycles of 15 s at 95°C, 30 s at 55°C, and 30 s at 72°C (ABI PRISM 7900 HT; Applied Biosystems). Expression (evaluated as fold change for each target gene) was normalized to glyceraldehyde-3-phosphate dehydrogenase (GAPDH, a housekeeping gene) following the well-established delta-delta method. All assays were performed in triplicate. In addition, a non-template control was included in the experiment to estimate DNA contamination of isolated RNA and reagents.

**Table 1:** Applied Primers for Real-time qPCR.

Genes	GenBank accession	Sense primers (5′ – 3′)	Anti-sense primers (5′ –3′)
CXCL1	NM_008176	CTGGGATTCACCTCAAGAACATC	CAGGGTCAAGGCAAGCCTC
IL-1β	NM_008361	GAAATGCCACCTTTTGACAGTG	CTGGATGCTCTCATCAGGACA
MCP-1	NM_011333	TAAAAACCTGGATCGGAACCAAA	GCATTAGCTTCAGATTTACGGGT
TNF-α	NM_013693	CAGGCGGTGCCTATGTCTC	CGATCACCCCGAAGTTCAGTAG
CTGF	NM_010217	GACCCAACTATGATGCGAGCC	TCCCACAGGTCTTAGAACAGG
TGF-β_1_	NM_011577	GAGCCCGAAGCGGACTACTA	GTTGTTGCGGTCCACCATT
Col1α2	NM_007743	AGCTTTGTGGATACGCGGAC	TAGGCACGAAGTTACTGCAAG
GAPDH	NM_008084	AGGTCGGTGTGAACGGATTTG	GGGGTCGTTGATGGCAACA

Abbreviations: CXCL1 - chemokine (C-X-C motif) ligand 1, IL-1β - interleukin 1 beta, MCP-1 - chemokine (C-C motif) ligand 2, TNF-α - tumor necrosis factor alpha, CTGF - connective tissue growth factor, TGF-β1 - transforming growth factor beta 1, Col1α2 - collagen type I alpha 2, GAPDH - glyceraldehyde-3-phosphate dehydrogenase.

### Data analysis

Statistical analysis was performed using the SPSS12.0 software package. Results were expressed as mean ± SE (standard error of mean). Differences among groups were tested by using One-Way ANOVA followed up with Tukey's test or t-test, as appropriate, and two-tailed p values are reported.

## Results

### Activation of mTOR signaling in kidney development and fibrogenesis

**Figure 1:**
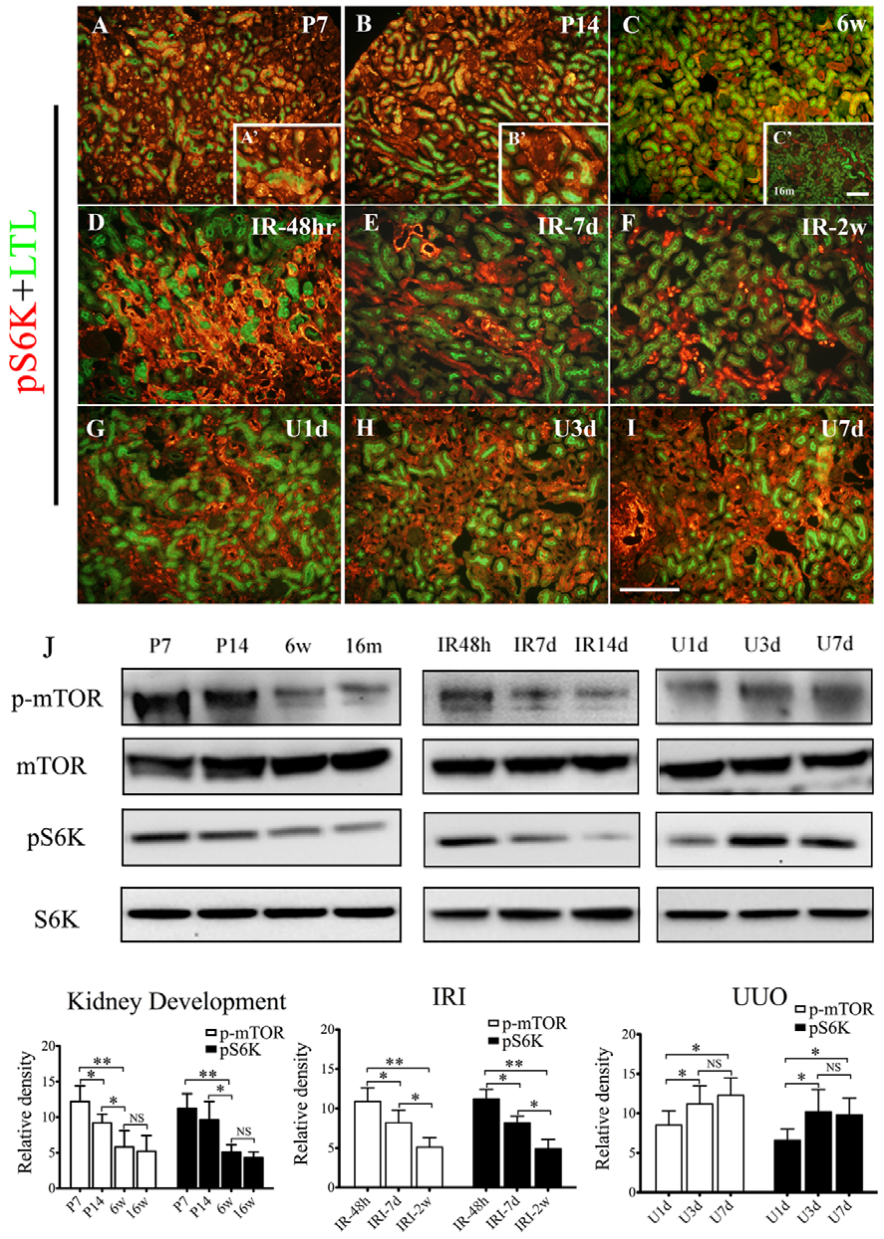
Assessment of immunoreactivity of mTOR signaling in normal and pathological kidneys. Kidney tissues derived from wide type C57BL/6J mice, moderate IRI and UUO mouse models were used for analysis. Animals were treated as described in Methods. A(A′)–C(C′): Costaining of pS6K (red) and LTL (green) in renal sections from post-natal day 1(P1, A: low-power and A′: high-power), post-natal day 7 (P7, B: low-power and B′: high-power), 6-week (6w, C) and 16-week mice (16w, C′). D–F: Representative costaining images of pS6K (red) and LTL (green) in kidney sections derived from moderate IRI mice, including 48-hour (IR-48 hr, D), 7-day (IR-7d, E) and 2-week (IR-2w, F) after operation. G–I: Representative costaining images of pS6K (red) and LTL (green) in kidney sections derived from UUO models, including 1-day (U1d, G), 3-day (U3d, H) and 7-day (U7d, I) post-obstruction. J: Western blot and quantitative analysis of p-mTOR and pS6K in developing kidneys (right panel), moderate IRI model (middle panel) and UUO model (left panel). n = 5 animals in each group. *P<0.05, **P<0.01, NS, no significance. Error bars represent S.E.

mTOR signaling highly activated, indicated by the expression of pS6K, in all kidney components (including renal parenchyma and interstitium) during the kidney development (Figure 1A(A′)–B(B′)), but declined to baseline in adult kidneys where its expression is mostly limited in small portion tubules (Figure 1C 6-week, Figure 1C′ 16-month). The protein amount of pS6K and p-mTOR in the kidneys derived from post-natal mice was significantly higher than adult kidneys (left column in Figure 1J), indicating mTOR signaling is essential for kidney development (i.e. cells growth) and might have some basic physiological functions (i.e. protein synthesis) in adult kidneys. The expression of pS6K can be significantly induced by either ischemic (Figure 1D and middle column in Figure 1J) or obstructive injury (Figure 1G and right column in Figure 1J) immediately. The activation of mTOR signaling returned to normal level along with the recovery of reversible kidney injury (Figure 1D–F and middle images in Figure 1J), but in irreversible obstructed nephropathy, activation of mTOR signaling kept increasing along with the progression of fibrosis (Figure 1G–I and right images in Figure 1J).

### Improvement of kidney fibrosis by administration of rapamycin

**Figure 2:**
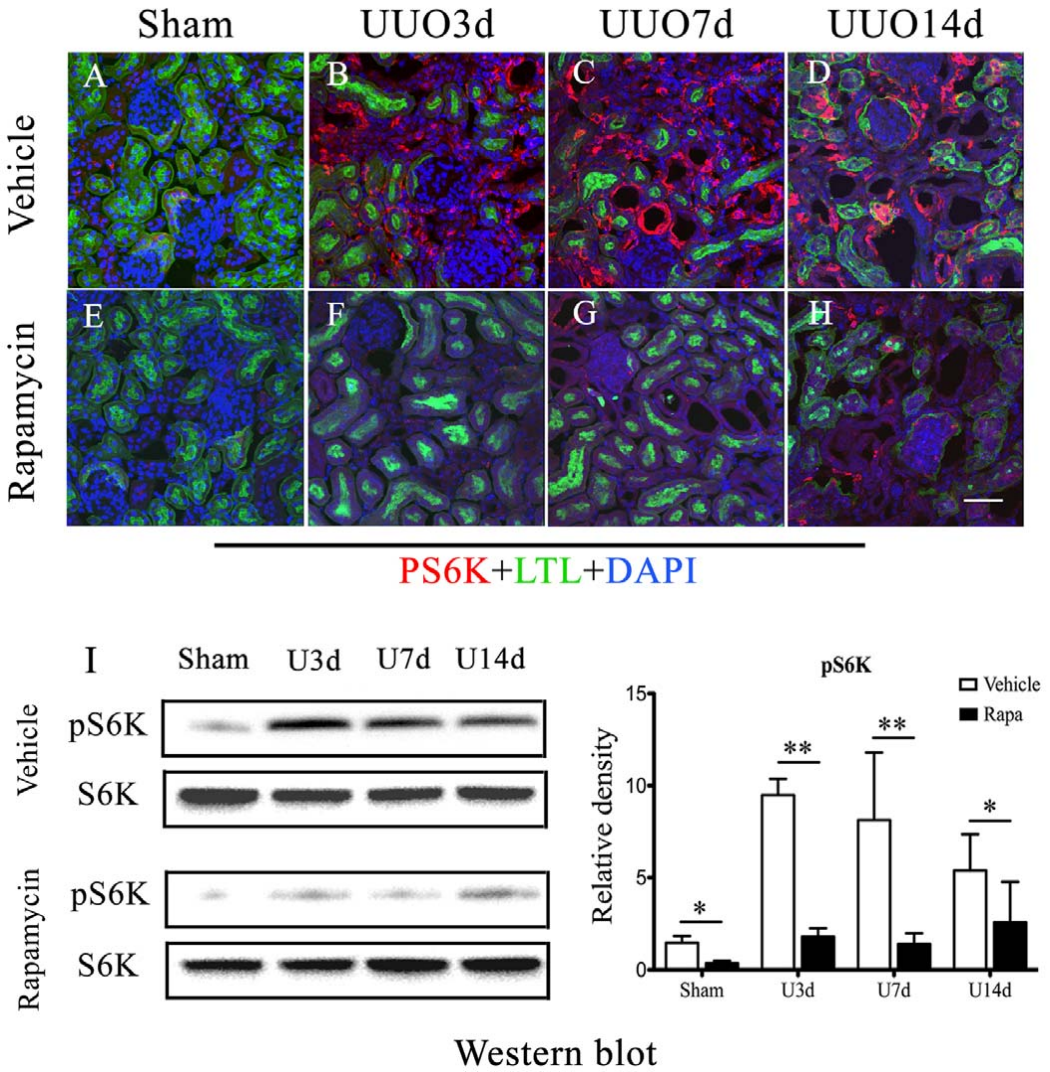
Inhibition of mTOR signaling by Rapamyicn in UUO mice. UUO mice received daily i.p. injections of rapamycin (2 mg/kg of body weight) or vehicle respectively, starting 1 day prior to operation and continuing until termination of the experiment. A–H: Representative co-staining images of pS6K (red) and LTL (green) in UUO kideny sections with (E–H) or without (A–D) rapamycin treatment, from day 0 (A, E), day 3 (B, F), day 7(C, G) and day 14(D, H). Nuclei were labeled with DAPI (blue). Scale bar = 50 µm. I: Representative western blot (panel left) and quantitative analysis (panel right) of pS6K expression in renal sections from vehicle-treated or rapamycin-treated UUO mice. *P<0.05, **P<0.01 vs. vehicle treated groups. Error bars represent S.E.

Expression of pS6K (red) and specific proximal tubule marker Lotus tetragonolobus lectin (LTL, green) were determined by immunofluorescent staining. Significant activation of mTOR signaling was observed in dilated tubules and interstitium of obstructed kidneys, but little in glomeruli (Figure 2A–D). Rapamycin significantly suppressed expression of pS6K in injured kidneys (Figure 2E–H). Quantitative assessment of pS6K by western-blot further confirmed that activation of mTOR signaling was highly induced after kidney injury and reached the peak around 7-day post-obstruction, whereas administration of rapamycin resulted in a significant inhibition of pS6K expression (Figure 2I).

**Figure 3:**
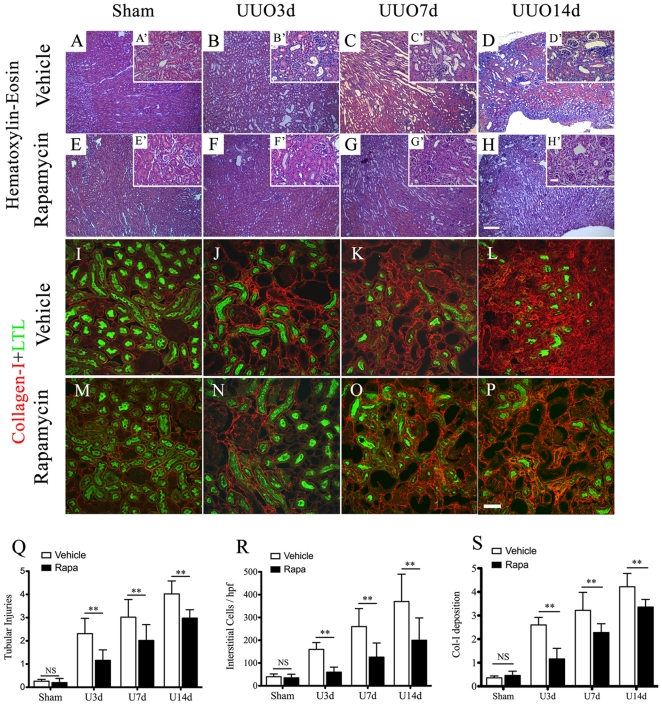
Rapamycin reduces tubulointerstitial injuries and collagen deposition in obstructed kidneys. Animals were treated as decribed above. A(A′)–H(H′): Representative sections of vehicle (A–D:low power, inserted A′–D′: high power) or rapamycin-treated (E-H: low power, inserted E′–H′: high power) kidney with hematoxylin-eosin staining. Scale Bar: 200 µm. I–P: Representative costaining sections of obstructed kidneys with vehicle (M–P) or rapamycin (I–L) treatment, using anti-collagen I (red) and anti-LTL (green) as primary antibodies. Scale bar: 50 µm. Q–S: Quantitative assessment of tubular injuries (Q), interstitial infiltrates (R) and collogen-I deposition (S) in kidneys derived from UUO mice with or without administration of rapamycin. **P<0.01 vs. vehicle treated groups, NS no significance. n = 5 animals for each group. Error bars represent S.E.

Immunohistochemical analysis with HE staining revealed progressive tubular dilation tubules atrophy, interstitial infiltrates and matrix deposition in mouse obstructed kidneys (Figure 3A–D, A′–D′: magnification of representative areas). Rapamycin markedly reduced these pathological changes. (Figure 3E–H and E′–H′). Co-staining of collagen-I and LTL showed that administration of rapamycin resulted in less collgen-I expression in the renal interstitium (Figure 3M–P), compared with the vehicle-treated group (Figure 3I–L). Quantitative analysis of kidney injury in UUO mice further confirmed that inhibition of mTOR signaling by rapamycin remarkably improved tubular injury (Figure 3Q), interstitial infiltrates (Figure 3R) and collogen-I deposition (Figure 3S).

### Rapamycin suppressed the proliferative activity of tubular and interstitial cells in obstructed kidneys

**Figure 4:**
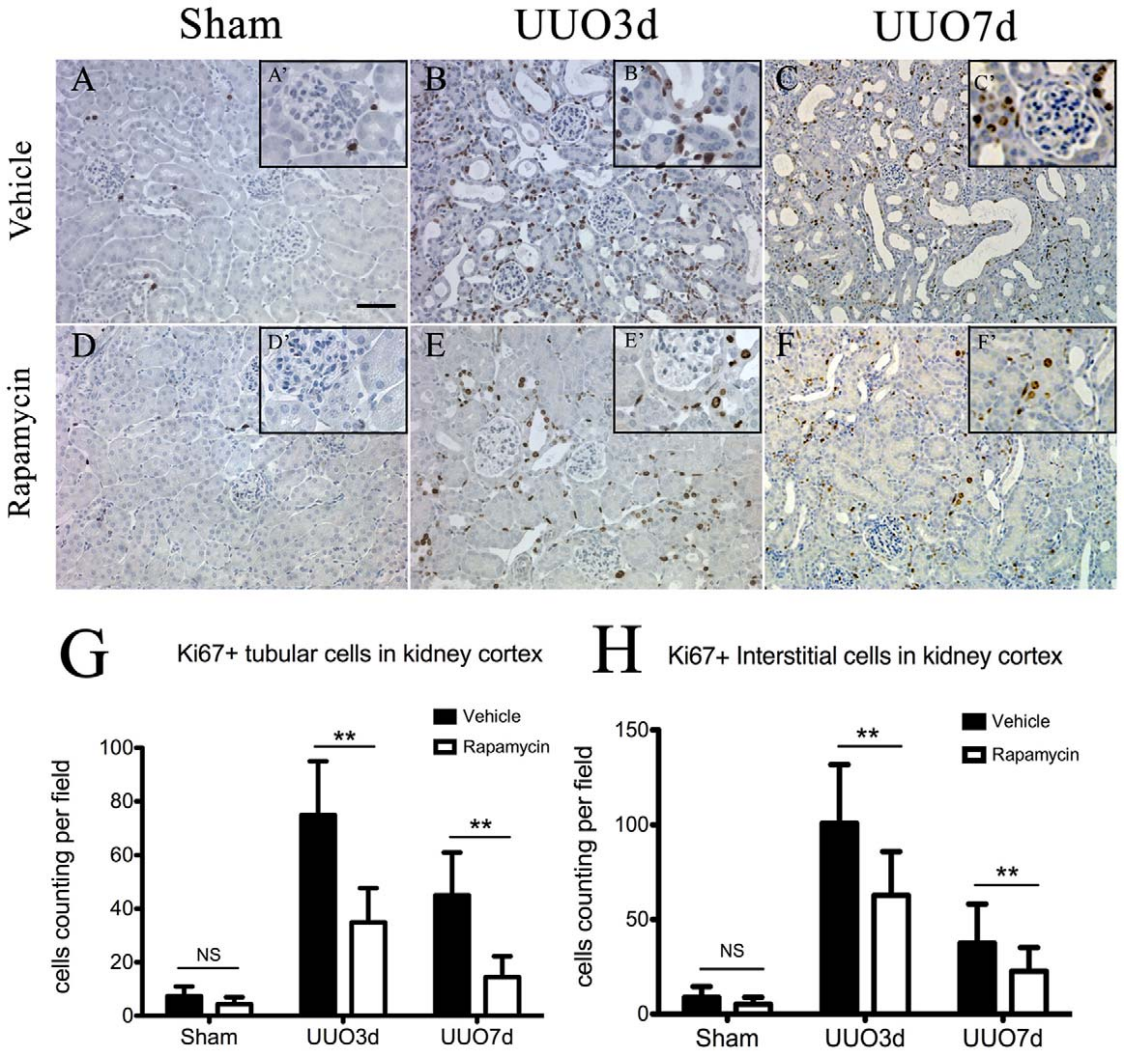
Rapamycin reduces proliferation in obstructed kidneys. Animals were treated as decribed in Methods. A(A′)–F(F′): Representative sections from kidneys with [D(D′)–F(F′)] or without [A(A′)–C(C′)] rapamycin treatment, using anti-ki67 for immunohistochemistry. Ki67 postive cells are labeled with brown staining. High-power micrographs are presented as inserted images (A′–F′). Scale bar: 50 µm. G–H: Quantitative analysis of Ki67+ tubular cells (G) and interstitial cells(H) in representative kidney sections. n = 5 animals for each group. **P<0.01 vs. vehicle-treated groups. NS no significance. Error bars represent S.E.

We analyzed the influence of rapamycin on the cells proliferation in fibrotic kidneys by immunohistochemical staining of anti-ki67 (brown). Only sporadic ki67-positive cells were observed in the normal kidneys of adult animals (Figure 4A–A′). However, a substantial increase of cell proliferation, including in tubules and interstium, was induced by ureter obstruction (Figure 4B–C, B′–C′), in accordance with the activation of mTOR signaling. Administration of rapamycin resulted in a significant decrease of cell proliferation, as well as tubulointerstitial involvement, in the obstructed kidneys (Figure 4D–F and D′–F′). Quantitative assessment of cell counting further confirmed that proliferation in both tubular (Figure 4G) and interstitial cells (Figure 4H) was significantly suppressed in rapamycin groups.

### The regulation of rapamycin on interstitial inflammatory cells during kidney fibrosis

**Figure 5:**
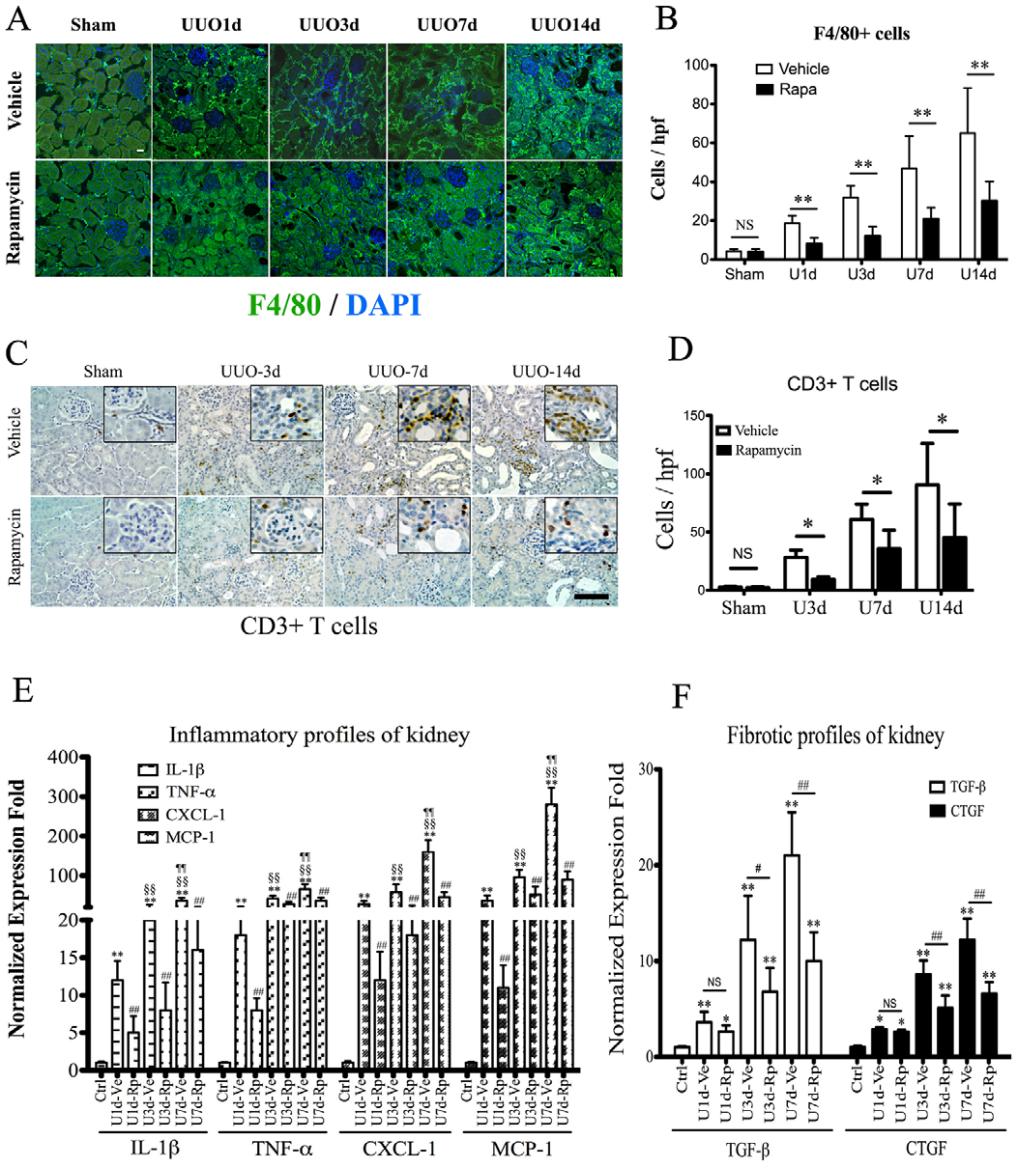
Rapamycin attenuates inflammatory responses in obstructed kidneys. A–B: Immunoflurescent staining (A) and quantitative assessment (B) of F4/80+ macrophages in kidney sections from UUO mouse models. anti-F4/80 (green) was used to label macrophages in kidney tissues, costaining with DAPI (blue). Scale bar: 20 µm. ** P<0.01 vs. vehicle treated groups. n = 5 animals in each group. C–D: Immunohistochemical staining (C) and quantitative assessment (D) of CD3+ T cells in kidney sections from UUO mice. anti-CD3 was used to label T cells in the kidneys, counterstained with hematoxylin (blue). Representative areas were magnified in the inserted images(C). Scale bar: 50 µm. *P<0.05 vs. vehicle treated groups. n = 5 animals in each group. E–F. Analysis of proinflammatory (E) and fibrotic (F) profiles of kidney tissues from UUO models, using quantitative realtime-PCR. IL-1β, TNF-α, CXCL-1 and MCP-1 were selected as pro-inflammatory chemokines for detection (E). **P<0.01, vs control group (sham operation); ^##^P<0.01, rapamycin vs. vehicle treated groups; ^§§^P<0.01 vs U1d-vehicle group; ^¶¶^P<0.01 vs U3d-vehicle group. n = 5 animals in each group. TGF-β and CTGF were selected as fibrotic cytokines for detection (F). *P<0.05, **P<0.01, vs control group (sham operation); ^#^P<0.05, ^##^P<0.01 rapamycin vs vehicle-treated group. NS no significance, n = 5 animals in each group. Error bars represent S.E.

Quantitative analysis by immunofluorescent and immunohistochemical staining revealed the marked infiltrates of F4/80+ macrophages (Figure 5A–B) and CD3+ T lymphocytes (Figure 5C–D) in obstructed kidneys since one day post obstruction, which aggravated along with the progression of interstitial fibrosis. Significantly, administration of rapamycin reduced the interstitial infiltrates of macrophasges and T lymphocytes in obstructed kidneys (Figure 5A–D). Further examination with real-time qPCR revealed that obstructed kidneys developed progressive inflammatory responses, indicated by elevating the expression of mutiple proinflammatory chemokines (IL-1β, TNF-α, CXCL-1 and MCP-1) in a time-dependent manner post-obstruction, which was dramatically reduced by rapamycin (Figure 5E). Real-time qPCR also revealed a lower expression of pro-fibrotic cytokines, including TGF-β_1_ and CTGF, in rapamycin treated groups (Figure 5F).

**Figure 6:**
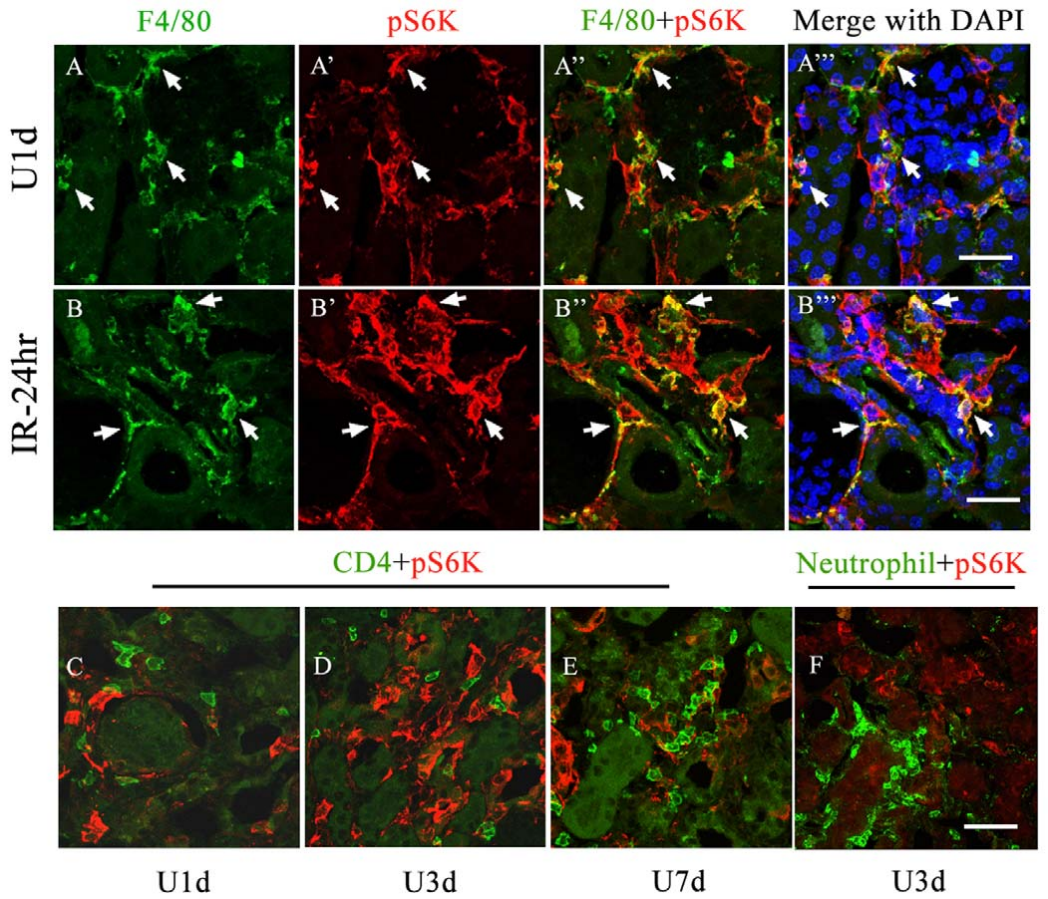
Activation profiles of mTOR signaling in the interstitial inflammatory cells. Kidney sections derived from either UUO or IRI models were analyzed by immunohistochemistry using antibodies against pS6K, F4/80, CD4 or anti-neutrophil. A–A′″ and B–B′″: Expression of pS6K (red) is immediately induced in F4/80+ macrophages (green) after kidney injury. Large portions of interstitial macrophages in obstructed kidney (A–A′″) and IRI kidney (B–B″) are co-stained with F4/80 and pS6K (indicated by arrwos). C–E: Co-staining of CD4 (green) and pS6K in obstructed kidneys. F: Costaining of anti-neutrophil and pS6K in obstructed kidneys. Scale bar: 20 µm.

In UUO and IRI mouse models, large portion of infiltrated F4/80+ macrophages in interstitium highly expressed pS6K on day-1 after the initiation of kidney injury (Figure 6A–A′″ and Figure 6B–B′″, representative cells indicated by white arrows). The obstructed kidneys also featured progressive infiltrates of CD4+ T cell and neutrophils, however, little expression of pS6K was observed in either CD4+ T cells (Figure 6C–E) or neutrophils (Figure 6F) in UUO mice. Therefore, the activation pattern of mTOR signaling in kidney inflammatory cells, revealed by immunoreactivity assessment, indicated that macrophages, instead of CD4+ T cells or neutrophils, may be direct targets of rapamycin in its anti-inflammation effects.

**Figure 7:**
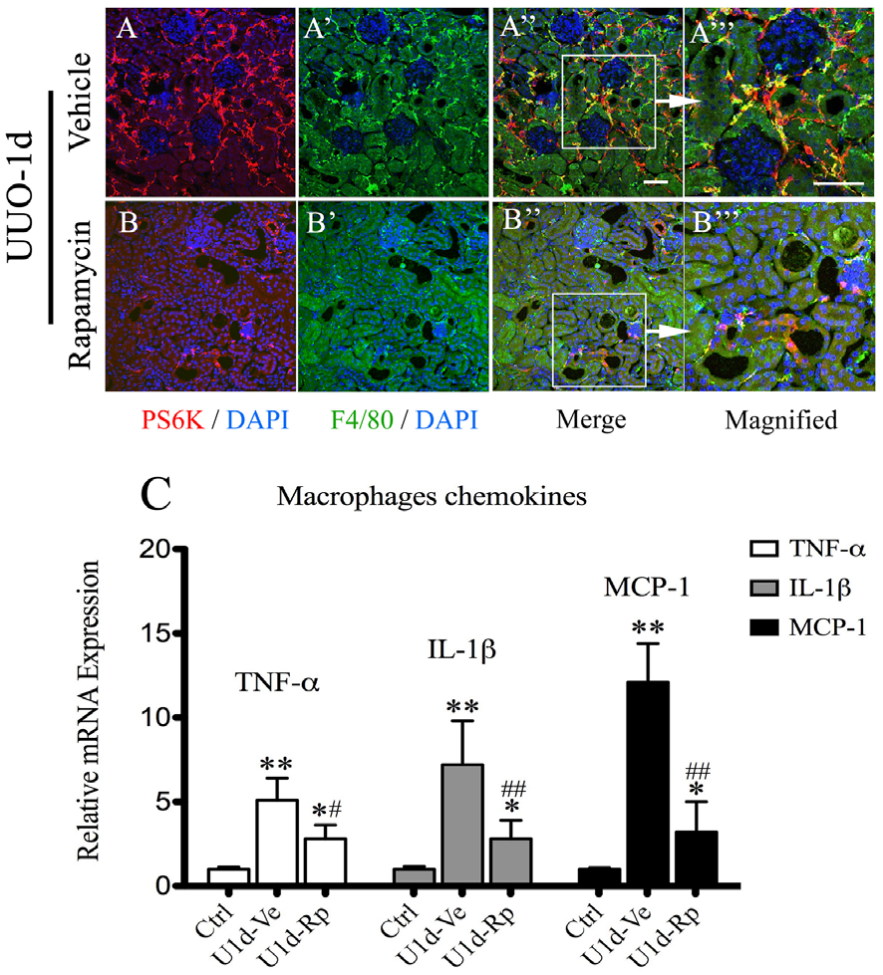
Rapamycin inhibits inflammatory activity of macrophages isolated from obstructed kidneys. A–B: Co-immunostainging images of kidney sections from day-1 post-obstruction with administration of rapamycin (A–A′″) or vehicle (B–B′″), using anti-pS6K (red), anti-F4/80 (green) and DAPI (blue) for immunofluorescent staining. Representative areas in A″ and B″ (indicated by white square) are magnified in A′″ and B′″, respectively. Scale bar: 50 µm. C. Assessment of inflammatory activity of isolated macrophages from obstructed kidney on day-1 post-operation. mRNA level of proinflammatory chemokines, including TNF-α, IL-1β and MCP-1, were determined by realtime-PCR. *P<0.05, **P<0.01, vs control group (sham operation); ^#^P<0.05, ^##^P<0.01 vs vehicle-treated group. n = 5 animals in each group. Ctrl: control group, U1d-Ve: 1-day post UUO operation with administration of vehicle, U1d-Rp: 1-day post UUO operation with administration of rapamycin. Error bars represent S.E.

To further confirm whether the mTOR signaling really regulates the activity of macrophages in the initiation of kidney fibrosis, we isolated macrophages from both vehicle (Figure 7A–A′″) and rapamycin-treated (Figure 7B–B′″) kidneys on day-1 post-obstruction and characterized their mRNA profiles of inflammatory chemokines, including IL-1β, TNF-α and MCP-1. Real-time qPCR analysis revealed that macrophages from rapamycin-treated groups presented much less inflammatory activity than vehicle groups (Figure 7C).

### The inhibition on the activation of myofibroblasts by rapamycin

**Figure 8:**
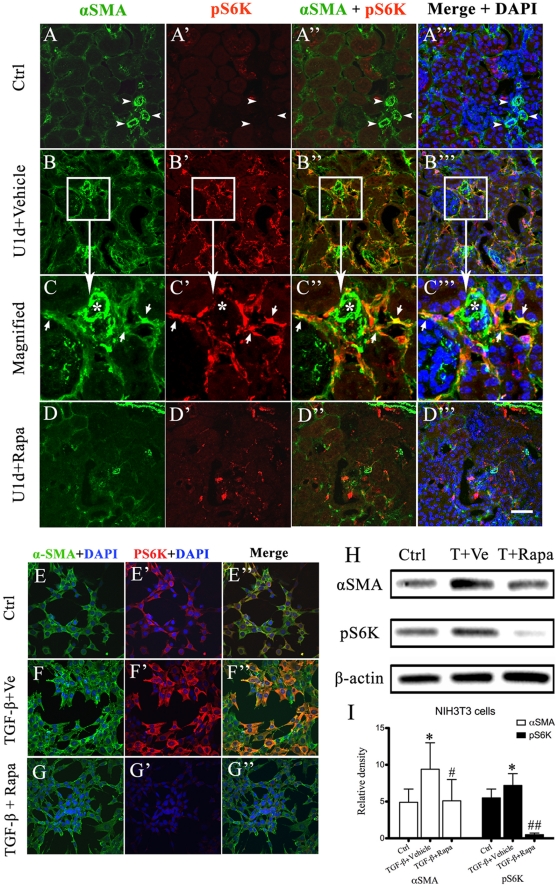
Rapamycin suppresses activation of mTOR signaling in active myofibroblasts. Immunofluorescent staining of kidney sections derived from UUO models on day-1 post-obstruction was performed, using anti-αSMA (green, A–D) and anti-pS6K (red, A′–D′). Images were merged (A″–D″) and co-labeled with DAPI (A′″–D′″). Representative areas in B–B′″ (white square) were magnified in C–C′″. Arrow heads (A–A′″) and asterisks (C–C′″) indicate arterioles in the kidneys Arrows indicate representative myofibroblasts expressing pS6K (C–C′″). Scale bar: 50 µm. NIH3T3 cells were cultured for 24 hours in the absence (E–E″) or presence of 20 ng/ml recombinant human TGF-β1 (F–F′″, G–G′″), with administration of vehicle (F–F′″) or rapamycin (G–G′″). The cells were stained for αSMA (green) and pS6K (red). DAPI was used to stain the nuclei. Magnification was 400×. (H–I): Representative Western blot and quantitative assessment for expression of αSMA and pS6K in NIH3T3 cells. β-actin was used in this experiment to control for equal protein loading. *P<0.05, **P<0.01 vs. control groups. ^#^P<0.05, ^##^P<0.01 vs. TGF-β+vehicle groups. Error bars represent S.E.

In normal kidney, αSMA expression was only observed in arteries and arterioles (Figure 8A–A′″ indicated by arrow-head). Low expression of pS6K was found in tubules and sporadic interstitial cells but little overlapped with the αSMA (Figure 8A–A′″). However, activation of fibroblasts and mTOR signaling were markedly induced on day-1 post ureteral obstruction, indicating by de novo expression of αSMA and pS6K within the interstitium (Figure 8B–B′). Merged images showed the co-expression of αSMA and pS6K in a large portion of interstitial myofibroblasts (Figure 8B″–B′″). Representative areas (Figure 8C–C′″) were maginified to further confirm the expression of pS6K in active myofibroblasts (white arrow), but not in arterial cells (asterisk). In rapamycin treated group, the expression of αSMA and pS6K in obstructed kidneys was significantly suppressed (Figure 8D–D′″). Given the pivotal role of TGF-β_1_ in kidney fibrosis, we further investigated the effect of rapamycin on TGF-β_1_-induced myofibroblast activation. NIH3T3 cells cultured in regular condition slightly expressed αSMA and pS6K (Figure 8E–E″). Stimulation of TGF-β_1_ in NHI3T3 cells resulted in marked expression of αSMA and pS6K (Figure 8F–F″), which was significantly suppressed by rapamycin (Figure 8G–G″). Western blot analysis further confirmed these changes (Figure 8H–I).

**Figure 9:**
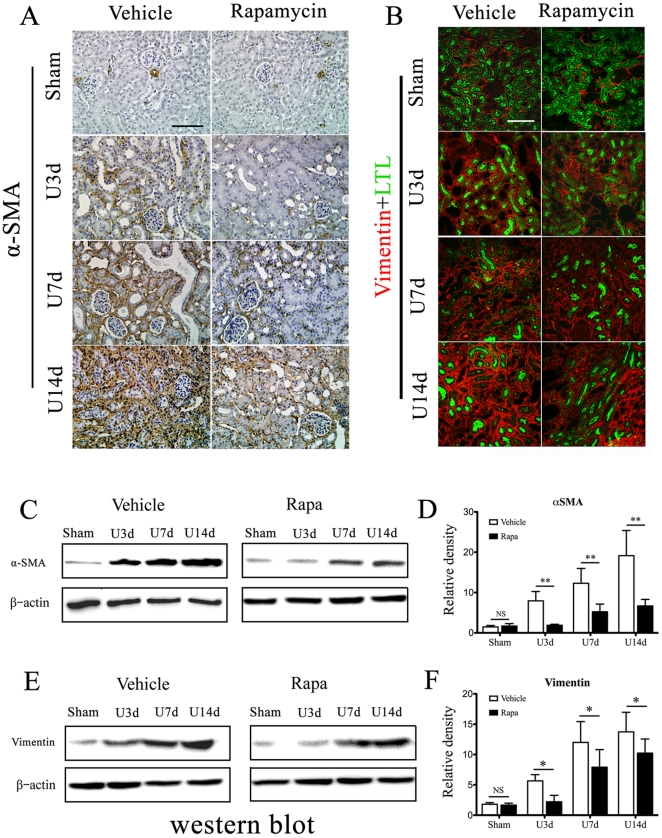
Assessment of myofibroblasts activation in obstructed nephropathy. Animals were treated as described in Methods and Materials. A. Representative sections of vehicle or rapamycin-treated kidneys stained with anti-αSMA by immunohistochemistry. Scale bar: 100 µm. B. Representative confocal images of vehicle or rapamycin-treated kidneys costained with anti-vimentin (red) and anti-LTL(green). Scale bar: 100 µm. C–F: Representative western blot and densitometric analyses for αSMA (C–D) and vimentin (E–F) expression in UUO kidneys. β-actin was used in this experiment to control for equal protein loading. Data were presented as mean ± S.E.M. n = 5 animals in each group. *P<0.05, **P<0.01 vs. vehicle-treated groups. NS no significance. Error bars represent S.E.

To further evaluate the effect of mTOR signaling on myofibroblasts transition, we quantify αSMA and vimentin expression in obstructed kidney using immunohistochemical staining and western blot analysis. In sham-operated kidney, a-SMA was found only in arteries and arterioles. However, along with the progression of interstitial fibrosis in obstructed kidneys, substantial accumulation of αSMA was found increasing within the interstium. The amount of αSMA deposition and renal histological changes were significantly ameliorated in rapamycin-treated kidneys (Figure 9A), indicating inhibiton of mTOR signaling markedly reduced myofibroblasts activation. Co-staining of vimentin (red) and LTL (green) further revealed the progressive accumulation of vimentin in the interstitium as a hallmark of fibrotic kidneys, which could be significantly attenuated by rapamycin (Figure 9B). Western blot of kidney lysates further confirmed that inhibition of mTOR signaling by rapamycin markedly reduced αSMA and vimentin production in UUO mice.

### The effect of rapamycin on fibrogenic phenotype of tubular epithelial cells

**Figure 10:**
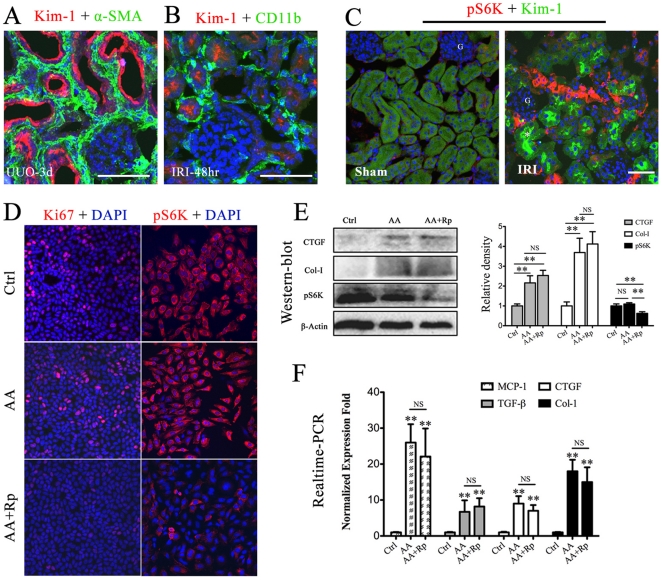
Rapamycin has little effect on the fibrogenic phenotype of tubular epithelial cells. HK2 cells or Animals were treated as described in Methods and Materials. A–C. Representative immunofluorescent costaining images of kidney sections derived from UUO mice or IRI mice, using anti-Kim-1 and anti-αSMA (A), anti-Kim-1 and anti-CD11b (B), anti-pS6K and anti-Kim-1 (C). Nuclei were labeled with DAPI (blue). Scale bar: 50 µm. D. HK2 cells were cultured for 48 hours in the absence or presence of aristolochic acid (AA), with or without administration of rapamycin. The cells were stained for Ki67 or pS6K. DAPI was used to stain the nuclei. E. Representative western blot and densitometric analyses for expression of CTGF, Collagen-I, and pS6K in cultured HK2 cells. β-actin was used in this experiment for equal protein loading control. Data were presented as mean ± SE. ** P<0.01, NS no significance. F. Assessment of proinflammatory and profibrogenic gene expression in culture HK2 cells. mRNA level of MCP-1, TGF-β, CTGF and Collagen-I were determined by realtime-PCR. ** P<0.01, NS no significance. Error bars represent S.E.

To determine whether the tubular epithelial cells remain potential targets of rapamycin in the progression of renal fibrosis, we studied the relationship between tubular fibrogenic activity and mTOR signaling. Kidney Injury Molecule-1 (Kim-1) was markedly up-regulated in either obstructed (Figure 10A) or ischemic kidneys (Figure 10B), where it localized to the apical surface of injured proximal tubule epithelial cells. These surviving epithelial cells, indicated by Kim-1 staining, were surrounded by abundant interstitial αSMA-positive myofibroblasts (Figure 10A) and CD11b-positive macrophages (Figure 10B), which revealed profibrogenic and proinflammatory roles of active epithelial cells after kidney injury. However, few Kim-1 positive epithelial cells co-expressed pS6K, although these tubules was surrounded by pS6K-positive intersitial cells (Figure 10C), indicating the mTOR signaling may play little role in the fibrogenic pathway conducted by active epithelial cells.

To further determine whether rapamycin could functionally affect the fibrogenic phenotype of epithelial cells, we examined the effect of rapamycin on the generation of profibrotic factors in cultured HK2 cells after exposure to aristotochic acid (AA). Immunocytochemistry confirmed that rapamycin markedly inhibited the proliferation and mTOR signaling in cultured HK2 cells (Figure 10D). AA treatment for 48 hr resulted in marked upregulation of CTGF and collagen-I in HK2 cells. Adminstration of rapamycin significantly inhibited pS6K expression but had little effect on the production of above fibrotic factors (Figure 10E). Real-time qPCR results confirmed that AA treatment induced the activation of fibrogenic and proinflammatory genes in HK2 cells, including TGF-β_1_, CTGF, Col1α2 and MCP-1. Administration of rapamycin did not improve the fibrogenic and inflammatory phenotype of HK2 cells, induced by AA treatment (Figure 10F).

## Discussion

Although the inhibitory effect of rapamycin on renal fibrosis has been reported in previous studies [1], [2], little was elucidated upon its cellular targets and regulatory mechanism. This study, for the first time, clarified that which cell types have mTOR activation in renal fibrosis and where rapamycin works on to protect the kidney.

Infiltration of inflammatory cells has long been established as an early and characteristic feature of renal fibrosis in virtually all situations [20]. We suggested that mTOR signaling might play an important role in the initiation and progression of kidney inflammation. To test this hypothesis, we characterized the activation profiles of mTOR signaling in different inflammatory cells types, including macrophages, CD4+ lymphocytes and neutrophils. Activation of mTOR and significant inflammatory response were induced in infiltrated macrophages post ischemic or obstructed injury, which could be significantly blocked by rapamycin (Figure 6 and Figure 7). Interestingly, little expression of pS6K was observed in either CD4+ lymphocytes or neutrophils in UUO mice, although rapamycin reduced the infiltrates of those inflammatory cells in the kidneys (Figure 5 and Figure 6). Therefore, the activation pattern of mTOR signaling in kidney inflammatory cells, revealed by immunoreactivity assessment, indicated that interstitial macrophages, instead of CD4+ T cells or neutrophils, might be direct targets of rapamycin. As multiple studies suggested that rapamycin presented a paradoxical aspect in regulating T cells immunobiology, depending on the subgroups of targeted T cell [21] and the conditions under which T cells are stimulated [22], it is difficult to conclude the role of rapamycin in regulating T cells in fibrotic kidneys based on our current observation. Furthermore, future experiments are also necessary to elucidate the role of mTOR signaling in the subgroups of macrophages, as macrophages present totally different phenotypes during kidney injury and repair progress, depending on the local inflammatory milieu [9], [23] and rapamycin might accordingly have different regulatory effects on them.

The presence of activated myofibroblasts is considered as a hallmark of kidney fibrosis in CKD [10]. In our study, colocalization of pS6K and αSMA in fibrotic kidneys revealed the activation of mTOR signaling in interstitial myofibroblasts, which was also supported by the experiment of TGF-β_1_ induced transition of fibroblasts into myofibroblasts (Figure 8). We further confirmed that the expression of αSMA and vimentin in obstructed kidney were significantly ameliorated in rapamycin-treated kidneys (Figure 9), indicating inhibiton of mTOR signaling markedly reduced myofibroblasts activation. Taken together, our studies revealed that rapamycin could ameliorate renal fibrosis by inhibiting the mTOR signaling in activated myofibroblasts.

Recently, emerging evidence indicated that tubular epithelial cells had an active role in the progression of renal fibrosis via generation of various proinflammatory and profibrotic factors, including cytokines, growth factors and matrix proteins [8], [24], [25]. To determine whether rapamycin protects the kidney from fibrosis partly by inhibiting the fibrogenic role of tubular epithelial cells, we labeled the surviving proximal tubules in both obstructed and ischemic kidneys, using anti-Kim-1, which has been widely identified as a sensitive and specific biomarker for injured proximal epithelial cells [26], [27]. After kidney injuries, significant infiltration of myofibroblast and macrophage was observed around the Kim-1 positive tubules (Figure 10 A–B), indicating surviving tubular epithelial cells recruited these effector cells and contributed to the interstitial fibrosis. However, little pS6K expression could be detected in the active tubular epithelial cells (Figure 10C), suggesting that mTOR signaling has not been activated in the fibrogenic epithelial cells. To further assess the effect of rapamycin on epithelial fibrogenesis, we established a cellular fibrotic model with HK2 cells secondary to AA exposure, which has been widely used to induce kidney interstitial fibrosis [28]–[30]. HK2 cells with AA treatment showed increased activity in pro-fibrogenesis and pro-inflammation, indicating by elevated protein and mRNA levels of TGF-β_1_, CTGF, Collagen-1 and MCP-1, but these levels were not lowered down by rapamycin (Figure 10E–F), providing further evidence that rapamycin does not directly block the fibrogenic activity of tubular epithelial cells during the progression of kidney interstitial fibrosis.

Activation of mTOR within the kidney has been reported in different kinds of kidney diseases, including acute ischemic injury [31], polycystic kidney disease [32], [33], diabetic nephropathy [34], [35] and other causes of progressive kidney disease [2], [36]. Although the overall effects of mTOR inhibitors on glomerular hypertrophy, interstitial inflammation and fibrosis, prove to be protective in CKD, the different function that mTOR signaling acts in acute and chronic kidney injury are significant [31], [37]. In this study, we observed the different expression pattern of mTOR signaling between reversible IRI and progressive fibrotic models (Figure 1), which indicated the induction of mTOR activation after kidney injury might therefore serve a dual purpose. Firstly, it is able to activate the innate immune effector cells at the early stage of injury, such as macrophages, which thereby clear apoptotic cells and debris at a site of tissue damage. Secondly, persistent mTOR activation in these effectors at an extended stage of injury, however, might be maladaptive by promoting chronic inflammation and ultimately renal fibrosis. Therefore, the balance of mTOR signaling activation could be a key to control the outcome of kidney injuries. Our data are in accordance with the current views that tightly balanced mTOR activity is required in kidney homeostasis and the role of rapamycin in kidney diseases is context dependent [1], [38].

In conclusion, this study confirmed the interstitial macrophages and myofibroblasts as the cellular targets of rapamycin for its protection of kidney fibrosis. Suppression of mTOR signaling in active macrophages and myofibroblasts leads to amelioration of kidney inflammation and fibrosis. A better understanding of the underlying mechanisms of mTOR signaling in renal fibrosis might help to find out a way to halt the fibrotic progression.
